# The Missing Evaluation at the End of GP's Consultation

**DOI:** 10.1155/2013/672857

**Published:** 2013-01-08

**Authors:** Maisa Kuusela, Paula Vainiomäki, Anni Kiviranta, Päivi Rautava

**Affiliations:** ^1^Department of Health Care and Social Services, City of Turku, PB 670, 20101 Turku, Finland; ^2^Department of Primary Health Care, Turku University Hospital, PB 52, 20521 Turku, Finland; ^3^Family Medicine, University of Turku, Lemminkäisenk 1, 20014 Turku, Finland; ^4^Säkylä Köyliö Municipal Primary Health Care Centre, Välskärintie 5, 27800 Säkylä, Finland; ^5^Public Health, University of Turku, Lemminkäisenkatu 1, 20014 Turku, Finland; ^6^Turku Clinical Research Centre, Turku University Hospital, PB 52, 20521 Turku, Finland

## Abstract

Evaluation at the end of a consultation is an element of a successful encounter. The doctor should inquire if patient's expectations were fulfilled and sum up the information given, the examinations performed, and the decisions made with the patient. This way the patient would be fully aware of what has been decided and that the problems and expectations of the patient had been taken into account. Twenty consultations of four general practitioners (GPs) in Finland were videotaped. The doctors were men and women, two of them had a long experience and two were trainees in general practice. The data (videotapes, questionnaires, and interviews) were analysed by multiple research methods with investigator and methodological triangulation. MAAS-Global Rating List was used as an assessment tool. The evaluation of the consultation was often missing or having shortages; only one-third was assessed to be better than doubtful. The assessments done by experienced GPs and the medical student were similar. According to the result of this study as well as the information in the current literature, doctors in all periods of their career should repeatedly be reminded about the importance of the evaluation at the end of the consultation.

## 1. Introduction

Patient centred approach, good patient-doctor relationship, and good communication skills are related to patient satisfaction, better outcome, and lower health care costs [1–3]. On the other hand, problems of patient-doctor communication are often the reason for complaints to medical regulatory authorities [4]. Poor information and lack of support by the health care personnel are related to a lack of adherence to treatment [5]. 

Some of the GP's core competencies are person/patient centred care, holistic modelling and comprehensive approach [6, 7]. There has been a discussion of the definition of patient centred care and, for example, patients' participation in the shared decision making [7–9]. The patients find it important to share their own view during the consultation, but, on the other hand, they expect their doctor to be an expert and an authority [10]. The patients do not always want to be involved in decision making, depending on the problem, age, or social class [11]. Doctors need skills to change their consultation style consistent with their patients' expectations and needs [8, 11]. 

According to some studies, longer consultations have been related to better communication and patient satisfaction [1, 7, 12]. And yet, the consultation length varies in different countries and is influenced by doctor and the country (i.e., the health care system) as well as by patient's gender and age and the number of problems discussed [13]. If the average length of the encounter is sufficient, the way it is in Sweden and Finland, the variation in length has no effect on the quality of communication [14, 15]. The factors to successful communication might be the GP's person and working style and the sufficient time reserved for patient's problem [12, 14]. 

Most of the patients come to the consultation with a particular agenda [16]. They might have several, unstructured problems, and only the minority of the patients expresses all items of their agenda during the consultation [17, 18]. GPs tend to bypass the patients' clues [19]. Patient centred consultation style encourages patients express their agendas [18]. The patients appreciate a competent and caring doctor who handles their problem seriously and with respect [20].

It is difficult for many, particularly elderly, patients, to remember GP's instructions and health information [21]. A carefully made evaluation of consultation supports the patient. It is an important and crucial element of a successful meeting between the patient and a doctor. At the end of the consultation, doctor should ask a general question if the patient's expectations were fulfilled and allow the patient to respond to this question. The doctor should sum up the basic information given, the examinations performed, and the decisions made with the patient. In this way the patient would be fully aware of what has been decided and that all the problems and expectations of the patient have been taken into account. Shared decision making and agreement between the patient and the doctor give support to the patient and provide better outcome [3].

In our study, we needed a practical, reliable, and valid method to assess GP's communication skills in the day to day practice. There are several methods to assess GP's communication skills, some of them are based on thorough analysis, and some are suitable only for educational purposes with simulated patients [22]. The methods measure different categories of communication skills and are variably practical [22, 23].

Video recording is a feasible and acceptable tool to assess GP's consultation. Both patients and GPs usually approve video recordings for research purposes. Video recordings have some effect on GP's and patient's performance, and some patients find videotaped consultations to be less confidential. Anyway, recordings are not likely to have negative effect on the quality of the consultation [24]. Nonverbal communication can be assessed and recordings can be analysed more deeply than in audiotaped or observed consultations.

The MAAS-Global method is a valid, reliable method which has been used to assess authentic GP's consultations and quality improvement [25, 26]. The MAAS-Global contains three sections: communication skills for each separate phase of consultation, general communication skills, and medical aspects. The method is concerned with the doctor's behaviour, not the patient's one. The items of communication skills are to be rated on a scale ranging from 0 up to 6: the best score (6), excellent, means that the GP could not have behaved better; the lowest (0) means that the item was not present at all [26]. 

The criterion corresponding to the rating “excellent” on item *evaluation of consultation *is as follows. At the end of the consultation doctor should ask a general question what the patient thinks or feels at that moment. This question needs not to concern any specific aspect of the consultation. The doctor should also check whether the patient's request for help has been adequately addressed and whether the patient has been offered perspective for the time being [26]. 

In our former study we compared patients' and GPs' assessments of the quality of consultations using questionnaires after consultation [15]. Similarly to other studies, both GPs and patients were very satisfied with the GPs communication skills [27, 28]. Patients are generally very positive about their GPs; some aspects of practice management are evaluated negative [29]. 

In this study we assessed GP's communication skills of the videotaped consultations with MAAS-Global method and compared the ratings with the information of the questionnaires and patient interviews. The aim of this study was to find a practical and economic tool to asses GP's communication skills. In this paper we focus on one characteristic of GP's communication skills, the evaluation at the end of the consultation.

## 2. Material and Methods

Altogether twenty consultations of four GPs in a primary health care centre in Turku, Finland, were videotaped. Our aim was to find two GPs, one male and one female with a long experience (over 10 years) in general practice and two trainees in general practice (<five years in general practise). To find voluntary GPs we sent an e-mail to all GPs (*n* = 100 on average) in one health care centre in Finland. We had no answers to this “first call.” When invited personally, it was quite easy to recruit experienced GPs, and a female trainee in general practice. It was more difficult to find a voluntary, less competent, male doctor—the fourth effort succeeded. We videotaped 4–6 consultations of each GP's normal day practice (for technical reasons every second encounter). The only exclusion criterion was patient's refusal. Only three patients declined to participate in the study. We did not ask the reason of refusal.

The informed consents were given, and the patients were interviewed in short before they went into the consultation room. In this before consultation interview we asked the patient's reason for the encounter, and if patient had any specific expectations concerning the consultation. The interview was briefly noted. The camera and digital audio recorder were installed in the consultation room before the consultation, and the camera was focused on the patient and the GP at the desk so that the examination desk was not seen (the discussion during the examination could be heard). The researcher was not present in the consultation room. The patients were interviewed (semistructured) once more after the consultation and it was audio recorded. In this after consultation interview we asked if patient's expectations were fulfilled and if something had remained unclear. The before consultation interview was made with 17 patients and the after consultation interview was made with 19 patients. The recordings and the interviews were transcribed. Both the patients and GPs separately completed a questionnaire concerning the satisfaction they experienced on the consultation. This questionnaire was also used in our earlier study [15]. 

Multimethod research with investigator and methodological triangulation was used in analysis. The videotapes were analysed by two researchers who were experienced GPs, both were specialists in general practice; they had worked years as GPs and as teachers in general practice and communication skills. The other one had also experience in the field of qualitative research. Deductive analysis was made according to the model of Maastricht University, the MAAS-Global Rating List in which the characteristics of consultation are assessed on a scale 0–6. The reliability of the assessment was guaranteed by parallel and also by consecutive evaluation. The characteristic “evaluation of the consultation” was also analysed by a third researcher, a fourth year medical student of a six years' basic medical education as a part of her medical studies. She analysed this item consecutively with one-month interval. These assessments were compared with the assessments of two other researchers. The final scores were reached at a mutual agreement after discussion in order to guarantee the reliability of the content analysis.

Two of the video recordings and translated (in English) transcriptions were sent for parallel assessment to Dr. Paul Ram, one of the developers of MAAS-Global method. To ensure the reliability of our assessments on expression of emotions, this item was also assessed by a psychologist who focused especially on the expression of emotions and on nonverbal communication.

One of the researchers (MK) analysed patients' questionnaires and prints of interviews to find out patients' problems, unfilled expectations, and other points which were not discussed during the encounter.

## 3. Results

The descriptions of the participating GPs are found in Table 1. We assessed the videotapings of 20 consultations (Table 2). The average age of the patients was 59 years and 60% of them were men. The average consultation time was 17.9 minutes (7–31 minutes). Four consultations were not counted in the mean, because the GPs and the patients left the room during the consultation (e.g., for operations). Consultation time included only the time when the patient was in the consultation room. In the GPs' opinion, patients brought up 2.4 problems in average (variation 1–7) during the encounter. 

**Figure 1:**
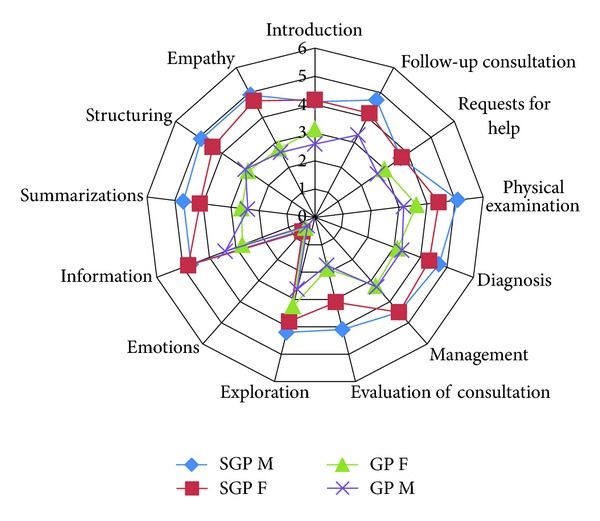
The mean scores of the items of communication skills (of each GP).

The mean scores of each GP are shown in Figure 1. In general, the scores of the items *physical examination*, *follow-up consultation*, *information, and diagnosis* were good. The lowest scores were given for items *emotions* and *evaluation of consultation*. The scores of the experienced GPs were higher than younger GPs in all items; the greatest differences were shown in *evaluation of consultation*, *summarisations*, *structuring*, and *empathy*.

All three researchers assessed the item *evaluation of the consultation* of 18 consultations with high mutual agreement (Table 2). Only in one case we could not reach agreement, mainly because of the consultation's unstructured form. 

*The evaluation of consultation* was quite often missing or having many shortages. Two-thirds of the consultations did not have an evaluation that had been assessed to have more than three (3) points out of six (6). 

The item *emotions *was missing in most of the consultations. The psychologist who assessed the expression of emotions agreed with the researchers. The expression of emotions was missing but, on the other hand, the patients' problems or complaints were not very emotive.

In the questionnaires, the quality of GP's communication skills was mostly assessed to be good or very good. Only in one consultation the patient assessed his GP's communication skills to be average, and in another consultation the GP assessed his/her own communication skills as average.

There were no crucial hidden agendas found in before or after consultation interviews. In one case the patient forgot to ask about tinnitus she had mentioned in the before consultation interview, and in another case patient remembered in the after consultation interview that he had forgotten to ask for a prescription. 

Doctor Paul Ram, one of the developers of the MAAS-Global method, agreed with our assessment concerning one of the two translated consultations, except for the items *request for help*, *evaluation of consultation*, and *emotions*, where we had 1-2 points' differences. The assessment of the other translated consultation on the basis of the text was not accomplished because the consultation was very long and unstructured.

## 4. Discussion

In this study, the GPs did not evaluate the consultation adequately with their patients. The evaluation of the consultation was missing quite often or having shortages; only one-third was assessed to be better than doubtful. The GPs performed well in the items *physical examination*, *follow-up consultation*, and *information and diagnosis*. The lowest scores were given for the item *emotions*. The scores of the experienced GPs were higher than younger GPs in all items; the greatest differences were shown in *evaluation of consultation*, *summarisations*, and *structuring and empathy*. The assessments done by the experienced GPs and a medical student were similar. The MAAS-Global method seems to be a useful tool for assessing GPs' communication skills.

The strength of this study can be considered to be in the multiple research methods and the combination of materials, the number of evaluators, and the use of external experts. We used a validated method and the reliability of the assessment was guaranteed by parallel and consecutive evaluation. Moreover, we could supplement our videotaped data with the questionnaire data. 

There are also few limits in the study. It is not possible to generalize these results in the Finnish primary health care because generalization is not in the scope of the qualitative study design. However, our material generated important information about GP's communication skills and the assessment of those skills. 

It is possible that we selected doctors who have better interaction skills than average doctors. It is unclear if the better skills of the competent specialists arise from the postgraduate specialist training, experience, or doctors' personal qualities. It is likely, however, that basic medical training provides insufficient ability to meet the patient, and these skills will be learned later, over the years in practice. 

The selection of patients was not very strong. The expression of emotions was missing in most of the consultations; it is possible that the problems of the three refusals could have been more difficult and more emotive to deal with. It is also possible that video recordings have inhibited the expression of emotions.

Video recording have some effects on both GPs' and patients' behaviour [24]. These effects are not always negative. One of our patients even suspected that it had positive impact on the course of the encounter. 

The MAAS-Global is a suitable tool for research use, and it is also useful in basic or postgraduate specialist training and in continuing medical education [25]. Although we found this method useful and our ratings were quite similar, we found it very time consuming. The method cannot be recommended for routine quality assessment in general practice without a proper familiarisation of the method. However, the video recordings of the consultations give valuable data for the doctors themselves to discover features and mannerisms in their work which they may not have even thought about.

The questionnaires and interviews did not provide as much information about the GP's communication skills as the MAAS-Global method. Both the GPs and the patients were very satisfied with the quality of the communication. The MAAS-Global method was capable of finding the differences in the GP's communication skills and the objectives for development.

MAAS-Global is a valid and reliable tool to assess GP's communication skills in the day to day practice [25–27, 30]. We modified the method and accepted minor differences in researchers' scores. Optimal ratings with MAAS-Global could be achieved only when the consultations are uncomplicated, in cases where the patient presents only one complaint and the consultation comprises all phases [26]. In this study, GPs estimated that patients presented 2.4 problems on the average. The researchers' opinion of the average number of problems was quite similar to GPs', although there were some differences in individual cases. Only five of our consultations fulfilled all the criteria of another MAAS-Global research [30], all the other had too many complaints, the consultations were too long, or the patients were too old. Anyway, the developer of MAAS-Global method (Dr.Paul Ram) agreed that it was a valid tool to assess our consultations.

Like in other studies, the GPs in our study did not adequately employ the evaluation of the consultation [25]. It is possible that some kind of summarisations and evaluation could have been found in the medical records. The original study design and research authorisation did not allow us to study the records. Anyway, the evaluations in the medical records do not benefit the patient; without the information it is not possible to ask, comment, or search agreement between the patient and doctor.

It is obvious that the perfect evaluation of the consultation, with respect for the patient's autonomy, promotes patient empowerment and self-management. The patient will be able to understand his symptoms better and will be more active and adherent to treatment. This is prerequisite for good health outcome, and therefore the evaluation of the consultation is an important item of GP's consultation skills.

In GPs' day to day work, the systematic assessment of communication skills seems to be rare in Finland. Communication skills can be improved with individual improvement activities based on performance assessment and feedback [25]. The evaluation and improvement of communication skills are an important part of doctors' professional development and must be included in continuing medical education. The importance of the careful evaluation of the consultation should be underlined.

## 5. Conclusion

Video recordings analysed with MAAS-Global method provide more information about the GP's communication skills than questionnaires and interviews.

According to the result of this study as well the information in the current literature, doctors in all periods of their career, from the start of basic medical education until the end of the continuous professional development, should repeatedly be reminded about the importance of the conclusion at the end of the consultation. GPs should pay attention to performing a patient centred and careful evaluation of each consultation to guarantee the best outcome of the consultation by promoting the patient empowerment.

## Figures and Tables

**Table 1:** Description of GPs.

	Gender	Age	Specialist GP/GP	Work experience as doctor (years)
SG PM	Male	57	Specialist GP	32
SG PF	Female	41	Specialist GP	16
TGP M	Male	31	Trainee in general practise	3
TGP F	Female	32	Trainee in general practise	5

**Table 2:** Descriptions of the consultations and scores for the item “the evaluation of the consultation” (S1: Researcher 1, experienced GP, S2: Researcher 2, experienced GP, S31: first scores of medical student, S32: second scores of medical student, and FS: final scores).

Descriptions of the patient and the main problems (researchers' opinion)	GP	Duration of the consultation (min)	S1	S2	S31	S32	FS	Comments
(1) Male, 74 years, several problems, follow-up consultation	SGP M	15	4	5	5	5	5	
(2) Female, 83 years, several problems, follow-up consultation	SGP M	28	4	4	5	5	4-5	With escort
(3) Male, 73 years, medical certificate, follow-up consultation of chronic diseases and medication	SGP M	19	4	3	4	5	4	
(4) Male, 69 years, diabetes, follow-up consultation	SGP F	15	3	4	5	4	4	
(5) Female, 25 years, diabetes, follow-up consultation	SGP F	28	4	4	5	5	4-5	
(6) Male, 37 years, diabetes, follow-up consultation	SGP F	22	3	3	3	3	3	
(7) Male, 57 years, follow-up consultation of hypertension, lipids, and diabetes	SGP M	23	5	5	6	5	5	
(8) Female, 25 years, back pain	SGP M	16	4	3	3	2	3	
(9) Male, 57 years, eczema and prescriptions	TGP F	14	4	4	2	2	3	
(10) Male, 60 years, coronary artery disease, follow-up consultation	TGP F	31	0	0	3	3	—	No agreement of final score
(11) Female, 22 years, flu	TGP F	7	2	3	3	3	3	
(12) Female, 48 years, eczema	TGP F	12	0	2	3	2	2	
(13) Male, 79 years, injured hand	SGP F	6*	—	—	—	—	—	Patient was sent to X-ray
(14) Male, 66 years, prolonged external otitis	SGP F	12*	3	3	2	3	3	Interruption (operation in operation room)
(15) Female, 66 years, trouble with knee	SGP F	17	2	2	4	3	2-3	
(16) Male, 74 years, several problems, follow-up consultation	TGP M	20	1	2	1	1	1	
(17) Female, 74 years, trouble with knee	TGP M	7*	—	—	—	—	—	Interruption (operation in operation room)
(18) Male, 41 years, rib pain, follow-up consultation	TGP M	7	0	1	1	0	0-1	
(19) Male, 80 years, several problems and prolonged cough	TGP M	11*	3	3	2	2	2-3	Interruption (X-ray and laboratory)
(20) Female, 68 years, follow-up consultation, new medication	TGP M	12	2	2	2	2	2	
